# Exploring PTSD in the General Population During the COVID-19 Pandemic: An Overview of Assessment Approaches and Global Prevalence Patterns

**DOI:** 10.1192/j.eurpsy.2026.10945

**Published:** 2026-07-31

**Authors:** P. Catapano, M. Luciano, S. Cipolla, G. Sampogna, F. Perris, F. Catapano, A. Fiorillo

**Affiliations:** Psychiatry, University of Campania “Luigi Vanvitelli”, Naples, Italy

## Abstract

**Introduction:**

The COVID-19 pandemic profoundly affected mental health worldwide. Post-traumatic stress disorder (PTSD) has been widely studied, but variability in assessment tools and cross-cultural differences remain insufficiently explored.

**Objectives:**

we aimed at: 1) comparing psychometric tools used to assess PTSD in adults during the pandemic; 2) investigating prevalence rates of PTSD in the general adult population during the pandemic, with a specific focus on cross-national differences; 3) identifying predictors and protective psychosocial factors for PTSD during the pandemic.

**Methods:**

A systematic search of PubMed, Scopus, and Web of Science was performed up to November 2024. Observational studies assessing PTSD in the adult general population during the COVID-19 pandemic were included. Extracted data covered prevalence, diagnostic instruments, predictors, and protective factors.

**Results:**

Forty-one studies from multiple regions were analyzed. Prevalence estimates varied widely, with the Impact of Event Scale-Revised yielding the highest rates and the Posttraumatic Stress Disorder Checklist the lowest. Mediterranean and Middle Eastern countries reported higher prevalence compared to Northern Europe. Risk factors consistently associated with PTSD included female sex, younger age, and social isolation. Protective factors, though less consistently reported, included older age as the most frequent finding, alongside perceived social support, health literacy, and—in some studies—transparent institutional communication. These resilience-related resources appeared to buffer symptoms regardless of infection status or lockdown measures.

**Conclusions:**

The pandemic substantially influenced PTSD outcomes in the community, though prevalence estimates were highly heterogeneous. Standardization of diagnostic tools is needed to enhance comparability across studies. Recognition of both risk and resilience factors may inform tailored interventions and guide public mental health strategies in future global crises.

**Disclosure of Interest:**

None Declared

